# RelA-Mediated BECN1 Expression Is Required for Reactive Oxygen Species-Induced Autophagy in Oral Cancer Cells Exposed to Low-Power Laser Irradiation

**DOI:** 10.1371/journal.pone.0160586

**Published:** 2016-09-15

**Authors:** Chih-Wen Shu, Hong-Tai Chang, Chieh-Shan Wu, Chien-Hsun Chen, Sam Wu, Hsueh-Wei Chang, Soong-Yu Kuo, Earl Fu, Pei-Feng Liu, Yao-Dung Hsieh

**Affiliations:** 1 Department of Medical Education and Research, Kaohsiung Veterans General Hospital, Kaohsiung, Taiwan; 2 Department of Surgery, Kaohsiung Veterans General Hospital, Kaohsiung, Taiwan; 3 Department of Dermatology, Kaohsiung Veterans General Hospital, Kaohsiung, Taiwan; 4 Radiation Oncology Department, Kaohsiung Veterans General Hospital, Kaohsiung, Taiwan; 5 Samwell Testing INC, Taipei, Taiwan; 6 Department of Biomedical Science and Environmental Biology, Kaohsiung Medical University, Kaohsiung, Taiwan; 7 Research Center of Environmental Medicine, Kaohsiung Medical University, Kaohsiung, Taiwan; 8 Department of Medical Laboratory Science and Biotechnology, School of Medical and Health Sciences, Fooyin University, Kaohsiung, Taiwan; 9 Department of Periodontology, School of Dentistry, National Defense Medical Center and Tri-Service General Hospital, Taipei, Taiwan; 10 Department of Biotechnology, Fooyin University, Kaohsiung, Taiwan; 11 Department of Stomatology, Kaohsiung Veterans General Hospital, Kaohsiung, Taiwan; ENEA Centro Ricerche Casaccia, ITALY

## Abstract

Low-power laser irradiation (LPLI) is a non-invasive and safe method for cancer treatment that alters a variety of physiological processes in the cells. Autophagy can play either a cytoprotective role or a detrimental role in cancer cells exposed to stress. The detailed mechanisms of autophagy and its role on cytotoxicity in oral cancer cells exposed to LPLI remain unclear. In this study, we showed that LPLI at 810 nm with energy density 60 J/cm^2^ increased the number of microtubule associated protein 1 light chain 3 (MAP1LC3) puncta and increased autophagic flux in oral cancer cells. Moreover, reactive oxygen species (ROS) production was induced, which increased RelA transcriptional activity and beclin 1 (BECN1) expression in oral cancer cells irradiated with LPLI. Furthermore, ROS scavenger or knockdown of RelA diminished LPLI-induced BECN1 expression and MAP1LC3-II conversion. In addition, pharmacological and genetic ablation of autophagy significantly enhanced the effects of LPLI-induced apoptosis in oral cancer cells. These results suggest that autophagy may be a resistant mechanism for LPLI-induced apoptosis in oral cancer cells.

## Introduction

Oral cancers consistently rank as one of the most common cancers worldwide, and more than 90% of oral cancers are oral squamous cell carcinomas (OSCCs) [1]. OSCC is one of the most common neoplasia and is frequently found on the tongue and on the buccal and gingival areas [2]. Standard treatments for early-stage oral cancer include surgery, radiation, and chemotherapy, which result in effective control of tumor progression. However, many patients receiving these treatments suffer severe cytotoxic side effects [3]. Low-power laser irradiation (LPLI) is the application of monochromatic coherent light at low energy levels, which can be used as a minimally invasive method for the treatment of tumors [4]. Previous results have indicated that LPLI at 810 nm selectively induces apoptosis in cancer cells but has little or no cytotoxic effect in normal cells [5]. High fluence LPLI (≧ 60 J/cm^2^) produces cytotoxic effects that interfere with the progression of the cell cycle and inhibit cell proliferation to control certain types of hyperplasia [6]. LPLI suppresses tumor growth and induces apoptosis in ASTC-a-1 human lung adenocarcinoma cells [7]. These results demonstrate that the antitumor effects of LPLI treatment involve in the induction of apoptosis [8,9], which is the preferred way to manage cancer.

Autophagy is an intracellular catabolic process by which the cell degrades long-lived proteins and damaged organelles, such as the endoplasmic reticulum, Golgi apparatus, and mitochondria via lysosomes for recycling as metabolic substrates to produce ATP under conditions of nutrient deprivation or stress [10]. Protective autophagy helps tumor cells to survive in conditions with increased metabolic demands by mitigating damage and recovering normal functions and protecting the cell from death [11]. Autophagy is induced in human cancer cells in response to laser irradiation [12]. Autophagy inhibitors increase the cytotoxicity of laser irradiation at 532 nm in glioma cells [12], suggesting that autophagy protects tumor cells from laser-induced stress. However, it has been widely reported that autophagy not only represents a cell survival mechanism but also directly contributes to death in stressed cells [13]. These results imply that autophagy may be essential in controlling the resistance/sensitivity of cancer cells exposed to LPLI therapy.

Reactive oxygen species (ROS) play a crucial role on apoptosis and autophagy in cells in response to laser irradiation. LPLI damages mitochondrial integrity and induces the production of a large amount of ROS [4,14]. Cytochrome c released from the mitochondria triggers a caspase 9/3 activation cascade, which appears to be largely mediated by direct ROS production in cells exposed to LPLI [5]. ROS, mainly H_2_O_2_, production also stimulates an increase in NF-κB activation in mouse embryonic fibroblasts treated with LPLI [14]. NF-κB can promote autophagy, but it can also inhibit autophagy in various cells under certain conditions [15] Moreover, RelA, a major member of the canonical NF-κB pathway, triggers BECN1 gene expression, which induces autophagy in T cells that have been stimulated with phorbol myristate acetate-ionomycin [16,17]. However, RelA has no effects on BECN1 mRNA expression in HeLa cells under heat shock conditions [18]. These results imply that the role of RelA in the modulation of autophagy may depend on the specific cells and the conditions under which they are stimulated. The specific roles of RelA and BECN1 on the process of autophagy in oral cancer cells irradiated with LPLI remain unclear.

Herein, we found that ROS production is important for the activation of RelA and for BECN1 expression, which in turn induces autophagy in oral cancer cells exposed to LPLI. This elevated autophagy leads to the development of a resistance to LPLI-induced apoptosis in oral cancer cells, implying that autophagy inhibitors may provide enhanced effects in LPLI-based therapy for OSCC.

## Materials and Methods

### Cell infection

Human OSCC cell lines, OECM-1 [19] and Ca9-22 [20], derived from gingival epidermoid carcinoma were originally provided by Dr. Ching-Liang Meng (National Defense Medical Center, Taipei, Taiwan) and kindly gifted by Dr. Hsiao Michael (Genomics Research Center, Academia Sinica, Taipei, Taiwan), respectively. Both OECM-1 and Ca9-22 were cultured in DMEM with 10% FBS, 100 μg/ml streptomycin, 100 U/ml penicillin, and 1% L-glutamine at 37°C with 5% CO_2_: 95% air. shRNAs against ATG7 (TRCN0000007584), SQSTM1 (TRCN0000007237), and RelA (TRCN0000014684) were obtained from The RNAi Consortium (TRC, Taiwan). The plasmids (2 μg) were transfected into HEK293FT cells (1 × 10^6^ cells) using 2 μl of Lipofectamine 2000 (Life Technologies, Inc.). The supernatant was harvested after 2 days, and the cell debris was removed for the infection of OECM-1 and Ca9-22 cells. The infected OECM-1 and Ca9-22 cells were selected with puromycin (3 μg/ml) for at least 10 days to obtain cells that were stably harboring shRNA. The knockdown efficiency in cells stably harboring shRNA against RelA, ATG7 and SQSTM1 was verified by immunoblotting.

### LPLI treatment

A soft tissue diode laser photocoagulation system (BioLase Technology, Inc.) coupled with an intraocular laser probe (which provided the endoprobe) was used for the soft tissue treatments. Irradiation experiments were performed in triplicate at room temperature using a continuous-wave 810 nm laser beam. The plastic covers were removed from the 96-well plates for the LPLI treatment. The laser power density used was 1 W/cm^2^. The cells were irradiated at this power density for 60 sec, which gave an energy density of 60 J/cm^2^.

### Confocal microscopy for GFP-MAP1LC3/SQSTM1 puncta and nuclear condensation

GFP-MAP1LC3 plasmids (0.15 μg) (Addgene) were transiently transfected into parental or shRNA stable Ca9-22 or OECM-1 cells with the X-tremeGENE transfection reagent (Roche Life Science) in 8-well glass Millicell EZ slides (Merck Millipore). The transfected cells were exposed to LPLI and recovered after 24 h and fixed with 4% paraformaldehyde (Sigma-Aldrich, Inc.). The fixed cells was also probed with an anti-SQSTM1 primary antibody (1:500, Enzo Life Sciences) followed by staining with an anti-rabbit secondary antibody conjugated to Alexia Fluor 568 (1:500; Roche Life Science). Images for GFP-MAP1LC3 and SQSTM1 puncta and colocalization were obtained at 40× magnification by confocal microscopy (LSM5 PASCALy). For apoptotic cells, the nucleus of each LPLI-irradiated cell was identified from the images of Hoechst 33342 stained cells. Hoechst 33342 staining was used to detect nuclear condensation and quantify apoptotic cells. Cells treated with autophagy inhibitors or inducers were used as negative or positive control, respectively.

### Autophagic flux measurement and immunoblotting

Parental or shRNA Ca9-22 and OECM-1 cells were treated with or without 20 μM CQ for 1 h prior to LPLI treatment. The recovered LPLI-treated cell lysates were used to detect the accumulation of MAP1LC3-II, a lipidated and membrane-bound form of MAP1LC3, by immunoblotting to determine autophagic flux [21]. For immunoblotting, the cells were briefly rinsed with PBS (Biological Industries, Kibbutz Beit-Haemek, Israel) and lysed with an lysis buffer [1% Triton X100, 50 mM Tris HCl at pH 7.5, 150 mM NaCl, 1 mM EDTA and a protease inhibitor cocktail (Roche Life Science)]. The proteins in the cell lysates were separated by SDS-PAGE and transferred onto nitrocellulose membranes. The membranes were incubated with primary antibodies against MAP1LC3 and ACTB (β-actin) (Sigma-Aldrich), SQSTM1 (BD Pharmingen), ATG7, RelA S536-P, and BECN1 (Cell Signaling Technology) overnight at 4°C. The proteins were probed with an HRP-labeled secondary antibody (Santa Cruz Biotechnology) and detected using an ECL reagent. The membranes were scanned and analyzed for protein expression level using a ChemiDoc XRS Imaging System (Bio-Rad Laboratories).

### ROS measurement

A hydrogen peroxide assay kit (BioVision, Inc., K265-200) was used to measure the concentration of H_2_O_2_. Briefly, the OxiRed Probe in the kit can react with H_2_O_2_ in the presence of Horse Radish Peroxidase (HRP) to produce red-fluorescent product, which can be detected by a reader with excitation wavelength at 535 nm and emission wavelength at 587 nm. Fifty μl of the reaction mixture containing OxiRed Probe and HRP was added to cells in each well of a 96-well plate. Cells treated with or without 10 mM N-acetyl-L-cysteine (NAC, a cell-permeable ROS scavenger, Sigma-Aldrich) for 1 h in 96-well microtiter plates (Costar, Corning) were used as controls. The H_2_O_2_ production in cells at 1 h after LPLI treatment was measured using fluorometry (Ex/Em = 535/587 nm) with a Fluoroskan Ascent FL reader (Thermo-Fisher Scientific).

### Luciferase assay for determining NF-κB activity

Luciferase reporter plasmids containing an NF-κB responsive promoter (Promega Corporation, pGL4.32) or a CMV constitutive promoter (Promega Corporation, pGL4.51) were transfected into oral cancer cells (8 × 10^3^ cells/50 μl) and seeded in each well of 96-well white plates overnight. The transfected cells were exposed to LPLI and recovered after 6 h. D-luciferin (200 μM) was then added to determine luminescence using a Fluoroskan Ascent FL reader (Thermo-Fisher Scientific). The cells with constitutive luciferase expression were used as controls for normalization.

### Cell viability and clonogenic assays

Oral cancer cells were seeded into 96-well white plates overnight prior to irradiation or treatment. The cells were lysed to determine cell viability with CellTiter Glo (Promega Corporation, G7571), which is a bioluminescent assay to measure cellular ATP level, an indicator of metabolically active cells. Alternatively, oral cancer cells were seeded at appropriate dilutions in 6-well plates overnight, followed by LPLI exposure. The cells were cultured for 2–3 weeks to form colonies. The colonies were fixed with paraformaldehyde (3.75% v/v), stained with crystal violet (0.25% w/v) and counted to determine the cytotoxic effects of LPLI on oral cancer cells.

### Spheroid cell culture and live/ dead assay

To mimic the effect of LPLI on oral cancer cells in vivo, the cells (4000 cells/well) were seeded into an ultra-low attachment, 96-well plate (Costar^®^, USA) and grown overnight to form spheroid cells. The cells were irradiated with LPLI in the presence or absence of CQ (20 μM) for 48 h. The spheroid cells were stained with Calcein AM (1 μM, green fluorescence) and Ethidium homodimer-1 (EthD-1, 2 μM, red fluorescence) (LIVE/DEAD^®^ Viability/Cytotoxicity Kit, ThermoFisher Scientific, L3324) for 30 minutes. The live (green) and dead [22] spheroid cells were imaged via fluorescence microscopy and quantitated using a Fluoroskan Ascent FL reader (Thermo Fisher Scientific) with excitation at 485 nm and emissions at 530 nm and 645 nm for calcein AM and EthD-1, respectively.

### Detection of apoptosis

For the apoptosis assay, the detached oral cancer cells treated with LPLI were washed with PBS and stained with propidium iodide (PI) and annexin V-FITC (Invitrogen, V13241) on ice for 10–15 min. The stained cells were analyzed using a FACScan flow cytometer (Becton Dickinson) and the number of apoptotic cells was quantified using FlowJo software (Tree Star). Alternatively, cells were seeded into 96-well white plate (2 × 10^4^ cells/well) for overnight and exposed to LPLI in the presence or absence of autophagy inhibitors. The cells were then recovered for 24 h and lyzed with Caspase-Glo 3/7 (Promega, G8091) to measure luminescent signal for 1 h. The net relative luminescence units (RLU) between time 0 and 1h were used to reflect caspase 3/7 activity in treated cells.

### Statistics

Data are reported as the mean S.E.M. from three independent experiments. Data were analyzed using Analysis of Variance (ANOVA) with Tukey’s post-HOC test. P-values less than 0.05 were considered significant (p-value ≦ 0.05 was considered significant (*), p-value ≦ 0.01 was considered highly significant (**), and p-value ≦ 0.001 was considered extremely significant (***).

## Results

### LPLI induces autophagic flux in oral cancer cells

**Fig 1:**
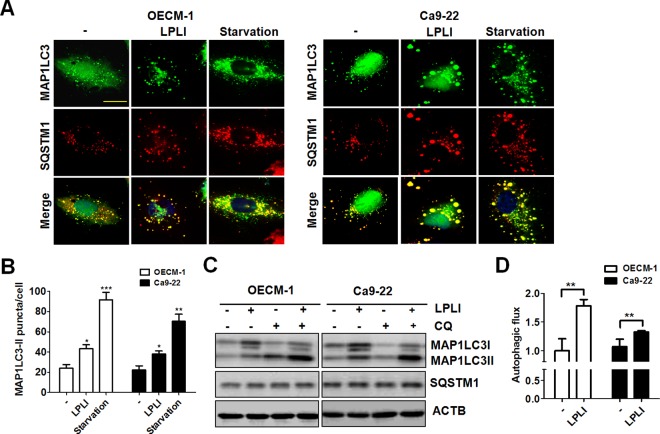
LPLI induces autophagic flux in human oral cancer cells. (A) OECM-1 or Ca9-22 cells harboring GFP-MAP1LC3 plasmids were irradiated with LPLI (810 nm, 60 J/cm^2^) and then recovered after 24 h. The recovered cells were fixed to examine GFP-MAP1LC3 and SQSTM1 puncta under confocal microscopy. Cells were starved with EBSS for 4 h as controls for autophagy induction. Scale bar: 10 μm. (B) The GFP-MAP1LC3 puncta were counted. (C) The recovered cells were lysed to determine the accumulation of MAP1LC3-II by immunoblotting. (D) MAP1LC3 degradation in cells with or without CQ (20 μM) was quantified to determine autophagic flux using ACTB as a normalization control. The data are expressed as the mean ± SEM from three independent experiments.

To determine the effects of LPLI on autophagy in oral cancer cells, OECM-1 and Ca9-22 oral cancer cells harboring GFP-MAP1LC3 were exposed to LPLI to observe the effect on GFP-MAP1LC3 puncta. The cells starved with Earle's Balanced Salt Solution (EBSS) were used as controls for autophagy induction (Fig 1A). GFP-MAP1LC3 puncta increased in OECM-1 and Ca9-22 cells after LPLI exposure (Fig 1A and 1B). SQSTM1, an MAP1LC3 binding protein that functions as an autophagy adaptor [23], was detected by immunostaining in these same cells. Similar to the patterns observed with GFP-MAP1LC3 puncta, an increase in SQSTM1 puncta was observed in LPLI-exposed cells (Fig 1A and 1B). This increase was mostly likely in autophagosomes because it was co-localized with GFP-MAP1LC3. Further, lipidated MAP1LC3-II turnover were used to monitor autophagic flux due to the MAP1LC3-II is degraded by autolysosome [21]. Thus, net MAP1LC3-II in cells with or without CQ was used to examine the effects of LPLI on autophagic flux. The level of MAP1LC3-II in LPLI-exposed cells, with or without CQ, was determined by immunoblotting (Fig 1C). The level of lipidated MAP1LC3-II was higher in LPLI-exposed oral cancer cells than in control cells. However, SQSTM1 levels were not altered in either the LPLI-exposed cells or the CQ-treated cells, suggesting SQSTM1 may be upregulated to restore the degraded protein level in cells under stress [24,25]. Therefore, the protein level of SQSTM1 may not be a sensitive indicator for determining autophagic activity in oral cancer cells exposed to LPLI. The autophagic flux in both oral cancer cell lines under LPLI exposure was quantified as previously reported [26] (Fig 1D). These results imply that LPLI induces autophagic flux in oral cancer cells.

### ROS trigger NF-κB-mediated BECN 1 expression for LPLI-induced autophagy

**Fig 2:**
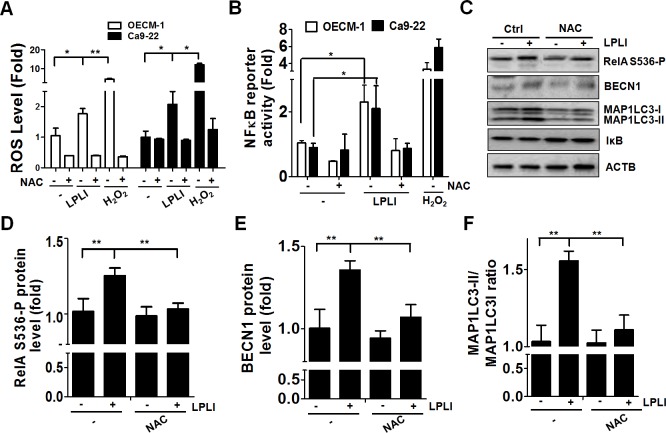
LPLI induces ROS-mediated RelA activation and BECN1 expression in human oral cancer cells. (A) OECM-1 (white) and Ca9-22 (black) cells were pretreated with (+) or without (-) 10 mM NAC prior to irradiation with LPLI (810 nm, 60 J/cm^2^). The ROS production in LPLI-treated cells was determined using an ROS assay kit and 100 μM H_2_O_2_ as a positive control. (B) OECM-1 (white) and Ca9-22 (black) cells transfected with the NF-κB-responsive luciferase vector were pretreated with or without 10 mM NAC for 1 h then irradiated with LPLI (810 nm, 60 J/cm^2^). The treated cells were recovered for 6 h and 200 μM D-luciferin was added to monitor luciferase activity. (C) OECM-1 or Ca9-22 cells pretreated with (+) or without (-) 10 mM NAC were irradiated with LPLI (810 nm, 60 J/cm^2^) then recovered for 24 h. The recovered cells were harvested for immunoblotting to determine the protein levels of phosphorylated RelA, BECN1, and MAP1LC3-II. Protein levels for (D) phosphorylated RelA and (E) BECN1, and (F) the MAP1LC3-II/I ratio were quantified using ACTB as a normalization control. The data are expressed as the mean ± SEM from three independent experiments.

**Fig 3:**
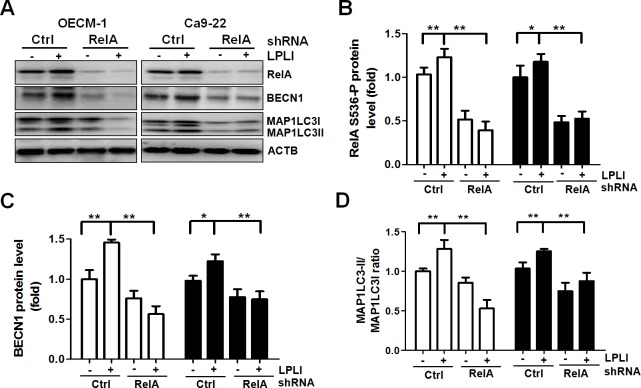
RelA stimulates BECN1 expression and the induction of autophagy in LPLI-exposed oral cancer cells. OECM-1 or Ca9-22 cells stably harboring scramble shRNA or shRNA against RelA were irradiated with (+) or without (-) LPLI (810 nm, 60 J/cm^2^) and recovered for 24 h. (A) The recovered cells were harvested for immunoblotting to determine protein levels of RelA, BECN1, and MAP1LC3. The protein levels for (B) RelA and (C) BECN1, and (D) the MAP1LC3-II/I ratio were quantified using ACTB as a normalization control. The results are expressed as the mean ± SEM from three independent experiments.

LPLI induces ROS production in various types of cancer cells [4,5]. To examine the involvement of ROS in LPLI-induced autophagy, we initially determined ROS production in OECM-1 or Ca9-22 oral cancer cells exposed to LPLI with an ROS fluorescent probe using H_2_O_2_ and the antioxidant NAC as controls (Fig 2A). The ROS production reached a plateau at 1 h (S1 Fig) and greater levels of ROS were produced in oral cancer cells exposed to LPLI compared to that in the control cells (Fig 2A). To determine whether LPLI-evoked ROS generation is essential for NF-κB activation, oral cancer cells were transfected with an NF-κB-responsive reporter vector and exposed to LPLI (Fig 2B). NF-κB transcriptional activity was increased in both OECM-1 and Ca9-22 oral cancer cells after irradiation, whereas NF-κB activity was decreased in the presence of NAC, indicating that ROS are required for the activation of NF-κB in oral cancer cells under LPLI exposure (Fig 2B). Moreover, the levels of phosphorylated RelA/p65 at Ser^536^ (RelA S536-P), a primary active component in the canonical NF-κB pathway, and BECN1 expression were elevated in LPLI-treated oral cancer cells. The elevation of RelA S536-P and BECN1 observed in cells after LPLI treatment was reduced by the addition of NAC (Fig 2C–2E). Similarly, MAP1LC3 expression and the ratio of MAP1LC3-II/I was increased after LPLI treatment but remained unchanged when cells were exposed to LPLI in the presence of NAC (Fig 2C and 2F). IκB levels were not altered in oral cancer cells treated with LPLI or NAC (Fig 2C). To further evaluate whether RelA activation is required for LPLI-induced BECN1 expression and autophagy, OECM-1 and Ca9-22 cells stably harboring shRNA against RelA were exposed to LPLI. RelA and BECN1 expression and the ratio of MAP1LC3-II/I in knocked down cells were determined by immunoblotting and quantified with ImageJ (Fig 3A–3C). Knockdown of RelA significantly suppressed the elevation in BECN1 and MAP1LC3 expression and the ratio of MAP1LC3-II/I in the cells exposed to LPLI (Fig 3C and 3D), suggesting that LPLI may trigger ROS production and activate RelA, thereby turning on BECN1 expression and inducing autophagy in oral cancer cells. However, it is known that the oxidative signal leads to a temporary inactivation of ATG4 at the site of autophagosome formation during the induction of autophagy [27]. In addition, silencing ATG4B increases autophagic flux in colorectal cancer cells [28]. Therefore, we examined whether ROS inactivates ATG4 to induce autophagy by measuring the proteolytic activity of ATG4 with the MAP1LC3-PLA_2_ reporter assay as previously reported [29,30] (S2 Fig). ATG4 activity was decreased in both OECM-1 and Ca9-22 oral cancer cells exposed to LPLI, whereas ATG4 activity was not recovered by the presence of NAC in LPLI-exposed cells. These results suggest that ROS may be required for RelA activation and BECN1 expression, instead of for ATG4 inactivation, for the induction of autophagy in oral cancer cells treated with LPLI.

### Autophagy inhibition increased LPLI-induced apoptosis in oral cancer cells

**Fig 4:**
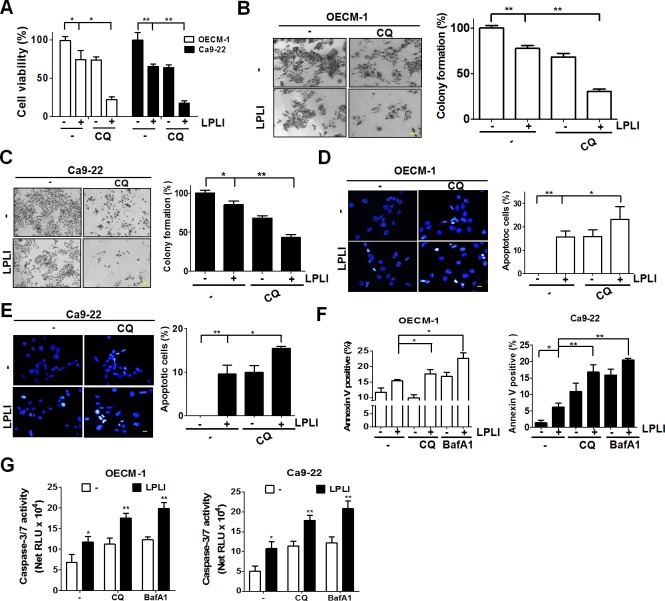
Autophagy inhibitors enhance LPLI-induced apoptosis in human oral cancer cells. OECM-1 or Ca9-22 cells were pretreated without (-) or with (+) 20 μM CQ for 1 h prior to irradiation with LPLI (810 nm, 60 J/cm^2^) and then recovered for 24 h. (A) The cell viability of the recovered cells was measured with CellTiter-Glo. The LPLI-treated (B) OECM-1 and (C) Ca9-22 cells were cultured for 14 days and stained with crystal violet to determine tumor colony formation. A representative sample of the results and the quantitative data are shown in the left and right panel, respectively. Scale bar: 100 μm. The recovered OECM-1 (D) and Ca9-22 (E) cells were fixed for staining with Hoechst 33342. The condensed nuclei of the cells were counted to quantify the number apoptotic cells. (F) The Ca9-22 and OECM-1 cells were pretreated with 20 μM CQ or 100 nM Baf A1 for 1 h prior to irradiation with LPLI and then recovered for 24 h. The recovered cells were harvested for PI/annexin V staining to verify the combined effects of LPLI and autophagy inhibitors on apoptosis in oral cancer cells. (G) The cells treated as panel F were lysed to measured caspase-3/7 activity with Caspase-Glo 3/7 luminescent assay. The results are expressed as the mean ± SEM from three independent experiments.

**Fig 5:**
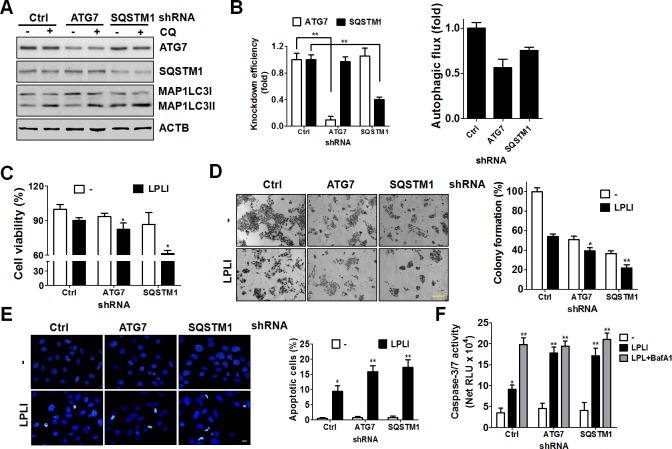
LPLI-induced apoptosis is elevated in autophagy-deficient oral cancer cells. (A) OECM-1 cells stably harboring shRNA against ATG7 or SQSTM1 were treated without (-) or with (+) 20 μM CQ prior to harvest. The harvested cells were used for immunoblotting to determine the protein levels of ATG7, SQSTM1 and MAP1LC3-II. (B) The knockdown efficiency of ATG7 and SQSTM1 in the cells was quantified using ACTB as a normalization control (left panel). The net protein levels of MAP1LC3-II between cells treated with or without CQ were used to determine autophagic flux as quantitated results in the right panel. (C) The recovered cells were accessed for cell viability with CellTiter-Glo. (D) The LPLI-treated cells were irradiated with LPLI and cultured for 14 days. Colony formation was accessed by staining with crystal violet. (E) The irradiated cells were fixed and stained with Hoechst 33342 to determine the number of apoptotic cells. Scale bar: 100 μm. The number of apoptotic cells is shown in the right panel. (F) The cells treated as panel E were lysed to measured caspase-3/7 activity with Caspase-Glo 3/7 luminescent assay. The results are expressed as the mean ± SEM from three independent experiments.

**Fig 6:**
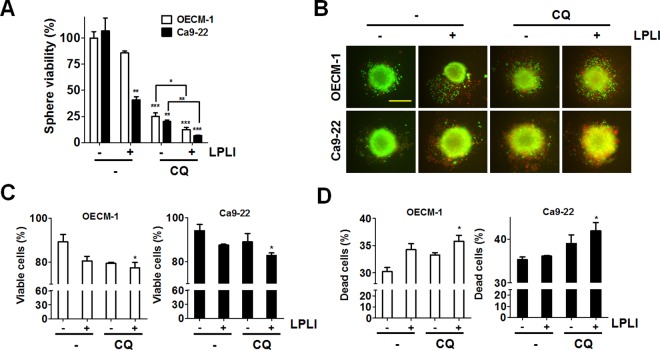
LPLI reduced tumor viability in spheroid culture. (A) OECM-1 or Ca9-22 cells were sphere cultured and then exposed to LPLI (810 nm, 60 J/cm^2^) in the presence or absence of CQ (20 μM) for 48 h. The spheres were lysed to measure ATP level for cell viability. (B) The viable and dead spheres as cultured and treated as (A) were imaged with LIVE (green)/DEAD (red) staining kit. Representative data are shown. Scale bar: 400 μm. (C) The green and (D) red fluorescence of the spheres as (B) was quantitated with a reader for the viable and dead cell population, respectively (n = 6). The quantified results are expressed as the mean ± SEM from 3 individual experiments. n.s., p > 0.05; *p < 0.05; **p < 0.01; ***p < 0.001.

Autophagy plays dual roles in the pathways supporting cell survival or death in cancer cells under different stresses. LPLI-irradiated oral cancer cells were examined in the presence of autophagy inhibitors to investigate the cytotoxic effects of 810 nm LPLI-induced autophagy. Cell viability and colony formation were significantly decreased in oral cancer cells treated with CQ prior to LPLI exposure compared with the effects observed in cells irradiated with LPLI alone (Fig 4A–4C). To precisely determine if apoptosis is involved in reduced viability, the number of cells with condensed nuclei, a common feature of apoptotic cells was counted. The apoptotic cells in CQ-pretreated OECM-1 (Fig 4D) and Ca9-22 (Fig 4E) cells were significantly increased. Furthermore, the number of annexin V/PI-positive Ca9-22 cells was increased in the cells exposed to LPLI in the presence of autophagy inhibitors (Fig 4F and S3 Fig). Likewise, autophagy inhibition enhanced the caspase-3/7 activity in LPLI-exposed oral cancer cells (Fig 4G). To more specifically determine the role of autophagy in LPLI-induced apoptosis, shRNAs against autophagy-related genes, including ATG7 and SQSTM1, were transfected into OECM-1 cells to generate autophagy-deficient cells. The knockdown efficiency of ATG7 and SQSTM1 was validated by immunoblotting (Fig 5A and 5B). The autophagic activity was quantitated according to MAP1LC3-II turnover in the cells (Fig 5B, right panel). Cell viability and colony formation were significantly reduced in autophagy-deficient cells after LPLI exposure compared with that observed in control cells with scrambled shRNA (Fig 5C and 5D). Moreover, apoptotic nuclear condensation was significantly increased in autophagy-deficient cells when exposed to LPLI (Fig 5E). Additionally, caspase 3/7 activity was increased in autophagy-deficient cells, whereas autophagy inhibitor did not enhance caspase-3/7 activity in the cells (Fig 5F). Taken together, these results indicate that autophagy inhibition may augment LPLI-induced apoptosis. Furthermore, the microenvironment of three-dimensional cell culture is relatively closed to complicated nature of tumor *in vivo* compared to two-dimensional cell culture [31]. Therefore, to mimic the effects of LPLI and autophagy inhibition in vivo, OECM-1 or Ca9-22 cells were cultured for spheroid formation and exposed to LPLI alone or combined with CQ (Fig 6A). Autophagy inhibition significantly decreased the ATP levels of the tumor sphere and enhanced the LPLI efficacy (Fig 6A). Similarly, the enhanced effects of autophagy inhibition on LPLI in the tumor sphere were confirmed via a LIVE/DEAD assay (Fig 6B–6D). These results supporting the idea that autophagy plays a cytoprotective role in oral cancer cells when exposed to LPLI. On the other hand, because ROS are important for cytoprotective autophagy in oral cancer cells exposed to LPLI, the cytotoxic effects of LPLI irradiation on oral cancer cells in the presence or absence of NAC was also examined (S4 Fig). NAC increased cell viability in oral cancer cells exposed to LPLI with and without CQ, suggesting that LPLI-induced ROS production may be required for both cytoprotective autophagy and apoptosis in oral cancer cells.

## Discussion

LPLI is known for its ability to cause cell death via the apoptotic pathway [32], a feature that has been used over the past decades for cancer therapy. Autophagy induced in cancer cells under irradiation provides a mechanism for cancer cell survival but can also promote cancer cell death [33,34]. The process that is induced might be cell and tissue specific and highly dependent on the gene expression profile regulating autophagy [34]. However, the missing links in the molecular mechanisms of LPLI-induced autophagy and the role of autophagy in LPLI-induced death in oral cancer are not fully understood. In this study, we present the following findings: first, LPLI induces ROS production and elevated RelA transcriptional activity in oral cancer cells. Second, RelA induces BECN1 expression, which induces autophagy in oral cancer cells exposed to LPLI. Third, this induced autophagy confers to LPLI resistance in oral cancer cells.

The value of laser-based anticancer therapy is limited [5], most likely because tumor cells are able to activate survival pathways that reduce the efficacy of LPLI. Previous research has highlighted the ability of autophagy to confer protection against external stresses, such as irradiation-induced cancer cell death, and to increase cell survival [35]. Numerous reports have indicated that autophagy is increased in photodynamic therapy (PDT)- and laser-induced cancer cell death [12,33]. Autophagy is frequently activated in radioresistant cancer cells [36], and the inhibition of autophagy sensitizes tumor cells to a wide spectrum of cancer therapies [35], including chemotherapy and irradiation therapy [3,12,35]. In line with previous reports, our current results showed that genetic and pharmacological ablation of autophagy facilitated LPLI-induced apoptosis, indicating that autophagy may reduce apoptosis and allow cells to survive under LPLI exposure. Notably, the autophagy inhibitor CQ is an anti-malarial drug and has been investigated as an anticancer drug or chemosensitizer in several clinical trials [37]. These results suggest that autophagy inhibition might be effectively used in combination with LPLI for OSCC therapy. Autophagy is induced in the salivary gland in response to radiation to balance proliferation and apoptosis [38]. Further in vivo studies are required to determine the effectiveness and potential side effects of a combined treatment with LPLI and CQ for OSCC patients.

ROS play a crucial role in several mechanisms of the induction of autophagy, including i) inactivation of ATG4 to eliminate the delipidation of MAP1LC3-II, and ii) the activation of AMPK and BECN1 [39]. Our present study showed that LPLI inhibited ATG4 proteolytic activity. The ROS scavenger NAC also attenuated ATG4 activity and did not restore the activity of ROS-inactivated ATG4. The inactivation of ATG4, which is not caused by ROS production, may play a role in LPLI-induced autophagy in oral cancer cells. Moreover, ROS can activate the IκB kinase (IKK) complex in certain types of cells, which in turn phosphorylates and causes the degradation of IκB, an inhibitor of NF-κB. In addition, ROS induce the phosphorylation of NF-κB at Ser-276 and Ser-536 to enhance the DNA-binding activity of RelA for the subsequent transcription of NF-κB target genes [40]. In agreement with these previous results, our data showed that the phosphorylation of RelA was increased in oral cancer cells exposed to LPLI, whereas LPLI-evoked ROS had no effects on IκB degradation (data not shown). These results suggest that LPLI-induced ROS may enhance the DNA binding activity of RelA and, therefore, enhance BECN1 expression for autophagy induction. On the other hand, many types of ROS may have different effects on LPLI-exposed cells. Previous report also shows O_2_^-•^ bust to damage cancer cells under high fluence LPLI [4], which suggest that H_2_O_2_ may trigger RelA/BECN1-mediated autophagy as a survival mechanism, whereas O_2_^-•^ confers to tumor suppressive effects in cancer cells under LPLI exposure. Nevertheless, the precise roles of different types of ROS on death or survival in oral cancer cells will require further study to clarify.

Crosstalk between the pathways for apoptosis and autophagy comes from shared upstream mediators in their core signaling mechanisms. Our present study indicated that the ROS scavenger NAC blocks the LPLI-induced protective autophagy and cell death in oral cancer cells. This result suggests that ROS act as initiators for triggering apoptotic and autophagic signaling in oral cancer cells when exposed to LPLI. It has been reported that the protein kinase ataxia-telangiectasia mutated [41] regulates autophagy [42] and apoptosis [43] under genotoxic conditions and oxidative stress. In addition, p53 has been identified as a regulator for switching between autophagy and apoptosis [22], indicating that the ATM/p53 pathway may be involved in the interplay between autophagy and apoptosis in LPLI-treated cells. By contrast, activated caspases can shut off the autophagic response by degrading the ATG proteins (i.e., VPS34, Beclin-1, ATG3, ATG5, and ATG7, AMBRA1) [44]. Conversely, autophagy inhibits apoptosis partly by degrading active caspase-8 or by preventing the activation of Bid by Beclin 1 [45]. Other genes, such as p27^kip1^, mTOR and death-associated protein kinas, have also been shown to be related to the crosstalk between autophagy and apoptosis [45]. Further study of the involvement of these core mediators in LPLI-induced autophagy/apoptosis in oral cancer cells is required to clarify the mechanisms involved, which could provide more effective treatment options for LPLI-based therapy.

In conclusion, the ROS/RelA/BECN1 axis plays a crucial role in LPLI-induced autophagy. This autophagy limits the antitumor effects of LPLI treatment on oral cancer cells. Our results provide the resistant mechanisms for LPLI-induced apoptosis in oral cancer cells.

## Supporting Information

S1 Fig The effects of LPLI on ROS production in human oral cancer cells. (TIF)

S2 Fig Effects of LPLI-evoked ROS on ATG4B proteolysis activity in human oral cancer cells. (TIF)

S3 Fig Autophagy inhibitors enhance LPLI-induced apoptosis in human oral cancer cells. (TIF)

S4 Fig ROS are involved in LPLI-mediated protective autophagy and cytotoxicity in human oral cancer cells. (TIF)

## Abbreviations

RelA: v-rel avian reticuloendotheliosis viral oncogene homolog A
BECN1: beclin 1
MAP1LC3: microtubule associated protein 1 light chain 3
SQSTM1: sequestosome 1
ATG: autophagy related gene
ACTB: beta-actin
ATM: ataxia-telangiectasia mutated
CQ: chloroquine
IKK: IκB kinase
LPLI: low-power laser irradiation
NAC: N-acetyl-L-cysteine
OSCC: oral squamous cell carcinomas
PDT: photodynamic therapy
PI: propidium iodide
ROS: reactive oxygen species

## References

[1] Jiang T, Liu G, Wang L, Liu H Elevated Serum Gas6 Is a Novel Prognostic Biomarker in Patients with Oral Squamous Cell Carcinoma. PLoS One. 2015; 10(7): e0133940 10.1371/journal.pone.0133940 26207647 PMC4514879

[2] Marocchio LS, Lima J, Sperandio FF, Correa L, de Sousa SO Oral squamous cell carcinoma: an analysis of 1,564 cases showing advances in early detection. J Oral Sci. 2010; 52(2): 267–73. 20587952 10.2334/josnusd.52.267

[3] Ahn MY, Yoon HE, Kwon SM, Lee J, Min SK, Kim YC, et al Synthesized Pheophorbide a-mediated photodynamic therapy induced apoptosis and autophagy in human oral squamous carcinoma cells. J Oral Pathol Med. 2013; 42(1): 17–25. 10.1111/j.1600-0714.2012.01187.x 22742535

[4] Wu S, Zhou F, Wei Y, Chen WR, Chen Q, Xing D Cancer phototherapy via selective photoinactivation of respiratory chain oxidase to trigger a fatal superoxide anion burst. Antioxid Redox Signal. 2014; 20(5): 733–46. 10.1089/ars.2013.5229 23992126 PMC3910666

[5] Liang WZ, Liu PF, Fu E, Chung HS, Jan CR, Wu CH, et al Selective cytotoxic effects of low-power laser irradiation on human oral cancer cells. Lasers Surg Med. 2015; 47(9): 756–64. 10.1002/lsm.22419 26395333

[6] Murayama H, Sadakane K, Yamanoha B, Kogure S Low-power 808-nm laser irradiation inhibits cell proliferation of a human-derived glioblastoma cell line in vitro. Lasers Med Sci. 2012; 27(1): 87–93. 10.1007/s10103-011-0924-z 21538143

[7] Wang F, Chen TS, Xing D, Wang JJ, Wu YX Measuring dynamics of caspase-3 activity in living cells using FRET technique during apoptosis induced by high fluence low-power laser irradiation. Lasers Surg Med. 2005; 36(1): 2–7. 15662635 10.1002/lsm.20130

[8] Chu J, Wu S, Xing D Survivin mediates self-protection through ROS/cdc25c/CDK1 signaling pathway during tumor cell apoptosis induced by high fluence low-power laser irradiation. Cancer Lett. 2010; 297(2): 207–19. 10.1016/j.canlet.2010.05.013 20579806

[9] Huang L, Jiang X, Gong L, Xing D Photoactivation of Akt1/GSK3beta Isoform-Specific Signaling Axis Promotes Pancreatic beta-Cell Regeneration. J Cell Biochem. 2015; 116(8): 1741–54. 10.1002/jcb.25133 25736682

[10] Mizushima N, Klionsky DJ Protein turnover via autophagy: implications for metabolism. Annu Rev Nutr. 2007; 27(19–40. 17311494 10.1146/annurev.nutr.27.061406.093749

[11] Mathew R, Karantza-Wadsworth V, White E Role of autophagy in cancer. Nat Rev Cancer. 2007; 7(12): 961–7. 17972889 10.1038/nrc2254 PMC2866167

[12] Krmpot AJ, Janjetovic KD, Misirkic MS, Vucicevic LM, Pantelic DV, Vasiljevic DM, et al Protective effect of autophagy in laser-induced glioma cell death in vitro. Lasers Surg Med. 2010; 42(4): 338–47. 10.1002/lsm.20911 20432283

[13] Ryter SW, Mizumura K, Choi AM The impact of autophagy on cell death modalities. Int J Cell Biol. 2014; 2014(502676. 10.1155/2014/502676 PMC3932252 24639873

[14] Chen AC, Arany PR, Huang YY, Tomkinson EM, Sharma SK, Kharkwal GB, et al Low-level laser therapy activates NF-κB via generation of reactive oxygen species in mouse embryonic fibroblasts. PLoS One. 2011; 6(7): e22453 10.1371/journal.pone.0022453 21814580 PMC3141042

[15] Trocoli A, Djavaheri-Mergny M The complex interplay between autophagy and NF-kappaB signaling pathways in cancer cells. Am J Cancer Res. 2011; 1(5): 629–49. 21994903 PMC3189824

[16] Copetti T, Bertoli C, Dalla E, Demarchi F, Schneider C p65/RelA modulates BECN1 transcription and autophagy. Mol Cell Biol. 2009; 29(10): 2594–608. 10.1128/MCB.01396-08 19289499 PMC2682036

[17] Copetti T, Demarchi F, Schneider C p65/RelA binds and activates the beclin 1 promoter. Autophagy. 2009; 5(6): 858–9. 19458474 10.4161/auto.8822

[18] Nivon M, Richet E, Codogno P, Arrigo AP, Kretz-Remy C Autophagy activation by NFkappaB is essential for cell survival after heat shock. Autophagy. 2009; 5(6): 766–83. 19502777 10.4161/auto.8788

[19] Chou CH, Yang NK, Liu TY, Tai SK, Hsu DS, Chen YW, et al Chromosome instability modulated by BMI1-AURKA signaling drives progression in head and neck cancer. Cancer Res. 2013; 73(2): 953–66. 10.1158/0008-5472.CAN-12-2397 23204235

[20] Yeh CC, Deng YT, Sha DY, Hsiao M, Kuo MY Suberoylanilide hydroxamic acid sensitizes human oral cancer cells to TRAIL-induced apoptosis through increase DR5 expression. Mol Cancer Ther. 2009; 8(9): 2718–25. 10.1158/1535-7163.MCT-09-0211 19737941

[21] Mizushima N, Yoshimori T, Levine B Methods in mammalian autophagy research. Cell. 2010; 140(3): 313–26. 10.1016/j.cell.2010.01.028 20144757 PMC2852113

[22] Livesey KM, Kang R, Vernon P, Buchser W, Loughran P, Watkins SC, et al p53/HMGB1 complexes regulate autophagy and apoptosis. Cancer Res. 2012; 72(8): 1996–2005. 10.1158/0008-5472.CAN-11-2291 22345153 PMC3417120

[23] Pankiv S, Clausen TH, Lamark T, Brech A, Bruun JA, Outzen H, et al p62/SQSTM1 binds directly to Atg8/LC3 to facilitate degradation of ubiquitinated protein aggregates by autophagy. J Biol Chem. 2007; 282(33): 24131–45. 17580304 10.1074/jbc.M702824200

[24] Fujita K, Maeda D, Xiao Q, Srinivasula SM Nrf2-mediated induction of p62 controls Toll-like receptor-4-driven aggresome-like induced structure formation and autophagic degradation. Proc Natl Acad Sci U S A. 2011; 108(4): 1427–32. 10.1073/pnas.1014156108 21220332 PMC3029726

[25] Son YO, Pratheeshkumar P, Roy RV, Hitron JA, Wang L, Zhang Z, et al Nrf2/p62 signaling in apoptosis resistance and its role in cadmium-induced carcinogenesis. J Biol Chem. 2014; 289(41): 28660–75. 10.1074/jbc.M114.595496 25157103 PMC4192515

[26] Liu PF, Cheng JS, Sy CL, Huang WC, Yang HC, Gallo RL, et al IsaB Inhibits Autophagic Flux to Promote Host Transmission of Methicillin-Resistant Staphylococcus aureus. J Invest Dermatol. 2015; 135(11): 2714–22. 10.1038/jid.2015.254 26134948 PMC4641007

[27] Scherz-Shouval R, Shvets E, Fass E, Shorer H, Gil L, Elazar Z Reactive oxygen species are essential for autophagy and specifically regulate the activity of Atg4. EMBO J. 2007; 26(7): 1749–60. 17347651 10.1038/sj.emboj.7601623 PMC1847657

[28] Liu PF, Leung CM, Chang YH, Cheng JS, Chen JJ, Weng CJ, et al ATG4B promotes colorectal cancer growth independent of autophagic flux. Autophagy. 2014; 10(8): 1454–65. 10.4161/auto.29556 24991826 PMC4203521

[29] Shu CW, Drag M, Bekes M, Zhai D, Salvesen GS, Reed JC Synthetic substrates for measuring activity of autophagy proteases: autophagins (Atg4). Autophagy. 2010; 6(7): 936–47. 10.4161/auto.6.7.13075 20818167 PMC3039740

[30] Shu CW, Madiraju C, Zhai D, Welsh K, Diaz P, Sergienko E, et al High-throughput fluorescence assay for small-molecule inhibitors of autophagins/Atg4. J Biomol Screen. 2011; 16(2): 174–82. 10.1177/1087057110392996 21245471

[31] Ernst A, Hofmann S, Ahmadi R, Becker N, Korshunov A, Engel F, et al Genomic and expression profiling of glioblastoma stem cell-like spheroid cultures identifies novel tumor-relevant genes associated with survival. Clin Cancer Res. 2009; 15(21): 6541–50. 10.1158/1078-0432.CCR-09-0695 19861460

[32] Huang L, Wu S, Xing D High fluence low-power laser irradiation induces apoptosis via inactivation of Akt/GSK3beta signaling pathway. J Cell Physiol. 2011; 226(3): 588–601. 10.1002/jcp.22367 20683916

[33] Kim J, Lim W, Kim S, Jeon S, Hui Z, Ni K, et al Photodynamic therapy (PDT) resistance by PARP1 regulation on PDT-induced apoptosis with autophagy in head and neck cancer cells. J Oral Pathol Med. 2014; 43(9): 675–84. 10.1111/jop.12195 24931630

[34] Palumbo S, Comincini S Autophagy and ionizing radiation in tumors: the "survive or not survive" dilemma. J Cell Physiol. 2013; 228(1): 1–8. 10.1002/jcp.24118 22585676

[35] Ko A, Kanehisa A, Martins I, Senovilla L, Chargari C, Dugue D, et al Autophagy inhibition radiosensitizes in vitro, yet reduces radioresponses in vivo due to deficient immunogenic signalling. Cell Death Differ. 2014; 21(1): 92–9. 10.1038/cdd.2013.124 24037090 PMC3857616

[36] Han MW, Lee JC, Choi JY, Kim GC, Chang HW, Nam HY, et al Autophagy inhibition can overcome radioresistance in breast cancer cells through suppression of TAK1 activation. Anticancer Res. 2014; 34(3): 1449–55. 24596393

[37] Kimura T, Takabatake Y, Takahashi A, Isaka Y Chloroquine in cancer therapy: a double-edged sword of autophagy. Cancer Res. 2013; 73(1): 3–7. 10.1158/0008-5472.CAN-12-2464 23288916

[38] Morgan-Bathke M, Lin HH, Ann DK, Limesand KH The Role of Autophagy in Salivary Gland Homeostasis and Stress Responses. J Dent Res. 2015; 94(8): 1035–40. 10.1177/0022034515590796 26092378 PMC4530390

[39] Poillet-Perez L, Despouy G, Delage-Mourroux R, Boyer-Guittaut M Interplay between ROS and autophagy in cancer cells, from tumor initiation to cancer therapy. Redox Biol. 2015; 4(184–92. 10.1016/j.redox.2014.12.003 25590798 PMC4803791

[40] Nowak DE, Tian B, Jamaluddin M, Boldogh I, Vergara LA, Choudhary S, et al RelA Ser276 phosphorylation is required for activation of a subset of NF-kappaB-dependent genes by recruiting cyclin-dependent kinase 9/cyclin T1 complexes. Mol Cell Biol. 2008; 28(11): 3623–38. 10.1128/MCB.01152-07 18362169 PMC2423290

[41] Prajna NV, Krishnan T, Mascarenhas J, Rajaraman R, Prajna L, Srinivasan M, et al The mycotic ulcer treatment trial: a randomized trial comparing natamycin vs voriconazole. JAMA Ophthalmol. 2013; 131(4): 422–9. 23710492 10.1001/jamaophthalmol.2013.1497 PMC3769211

[42] Liang N, Jia L, Liu Y, Liang B, Kong D, Yan M, et al ATM pathway is essential for ionizing radiation-induced autophagy. Cell Signal. 2013; 25(12): 2530–9. 10.1016/j.cellsig.2013.08.010 23993957

[43] Luo L, Huang W, Tao R, Hu N, Xiao ZX, Luo Z ATM and LKB1 dependent activation of AMPK sensitizes cancer cells to etoposide-induced apoptosis. Cancer Lett. 2013; 328(1): 114–9. 10.1016/j.canlet.2012.08.034 22960274 PMC3521637

[44] Wu H, Che X, Zheng Q, Wu A, Pan K, Shao A, et al Caspases: a molecular switch node in the crosstalk between autophagy and apoptosis. Int J Biol Sci. 2014; 10(9): 1072–83. 10.7150/ijbs.9719 25285039 PMC4183927

[45] Nikoletopoulou V, Markaki M, Palikaras K, Tavernarakis N Crosstalk between apoptosis, necrosis and autophagy. Biochim Biophys Acta. 2013; 1833(12): 3448–59. 10.1016/j.bbamcr.2013.06.001 23770045

