# Supplementary figures and images for: RelA-Mediated BECN1 Expression Is Required for Reactive Oxygen Species-Induced Autophagy in Oral Cancer Cells Exposed to Low-Power Laser Irradiation
Source: PLoS One. 2016 Sep 15;11(9):e0160586. doi: 10.1371/journal.pone.0160586 (PMC5025201; doi:10.1371/journal.pone.0160586)

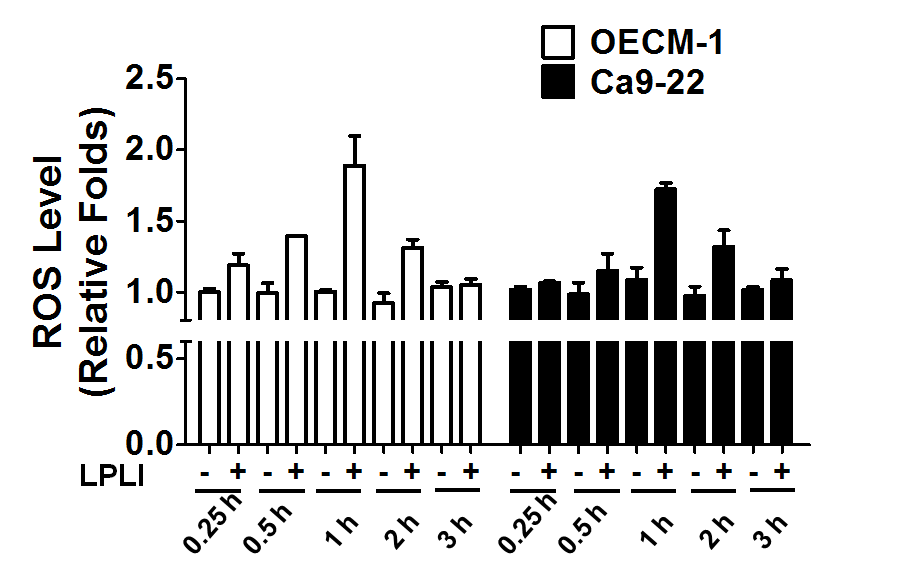

Supplement: S1 Fig — (TIF) [file pone.0160586.s001.tif]

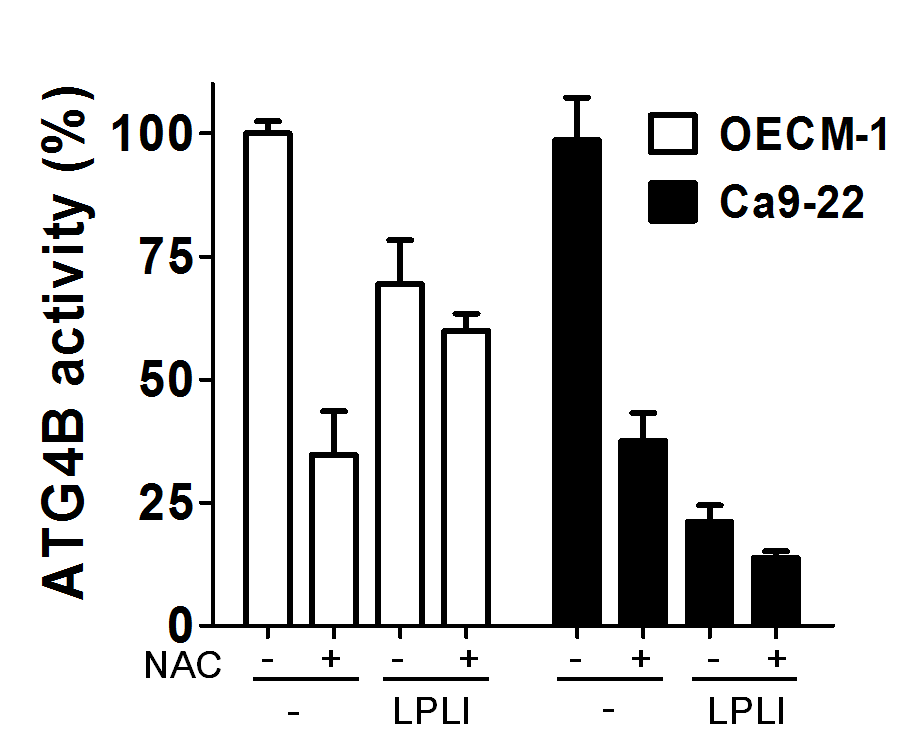

Supplement: S2 Fig — (TIF) [file pone.0160586.s002.tif]

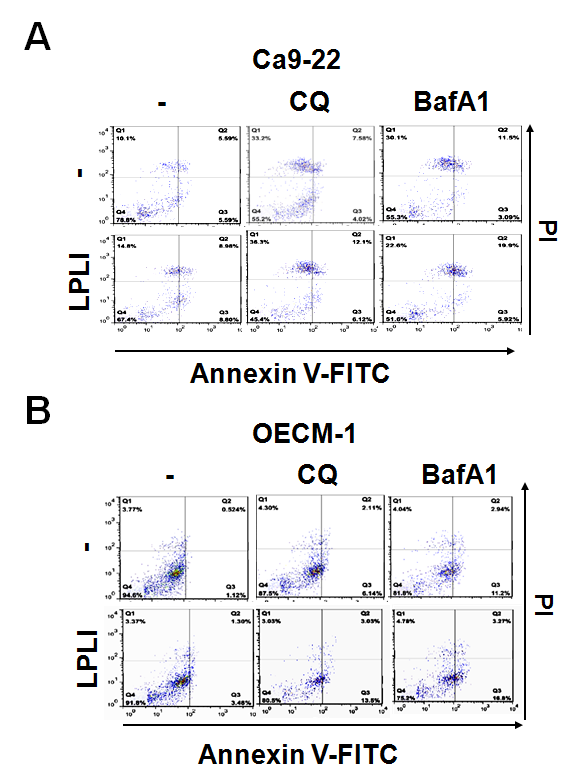

Supplement: S3 Fig — (TIF) [file pone.0160586.s003.tif]

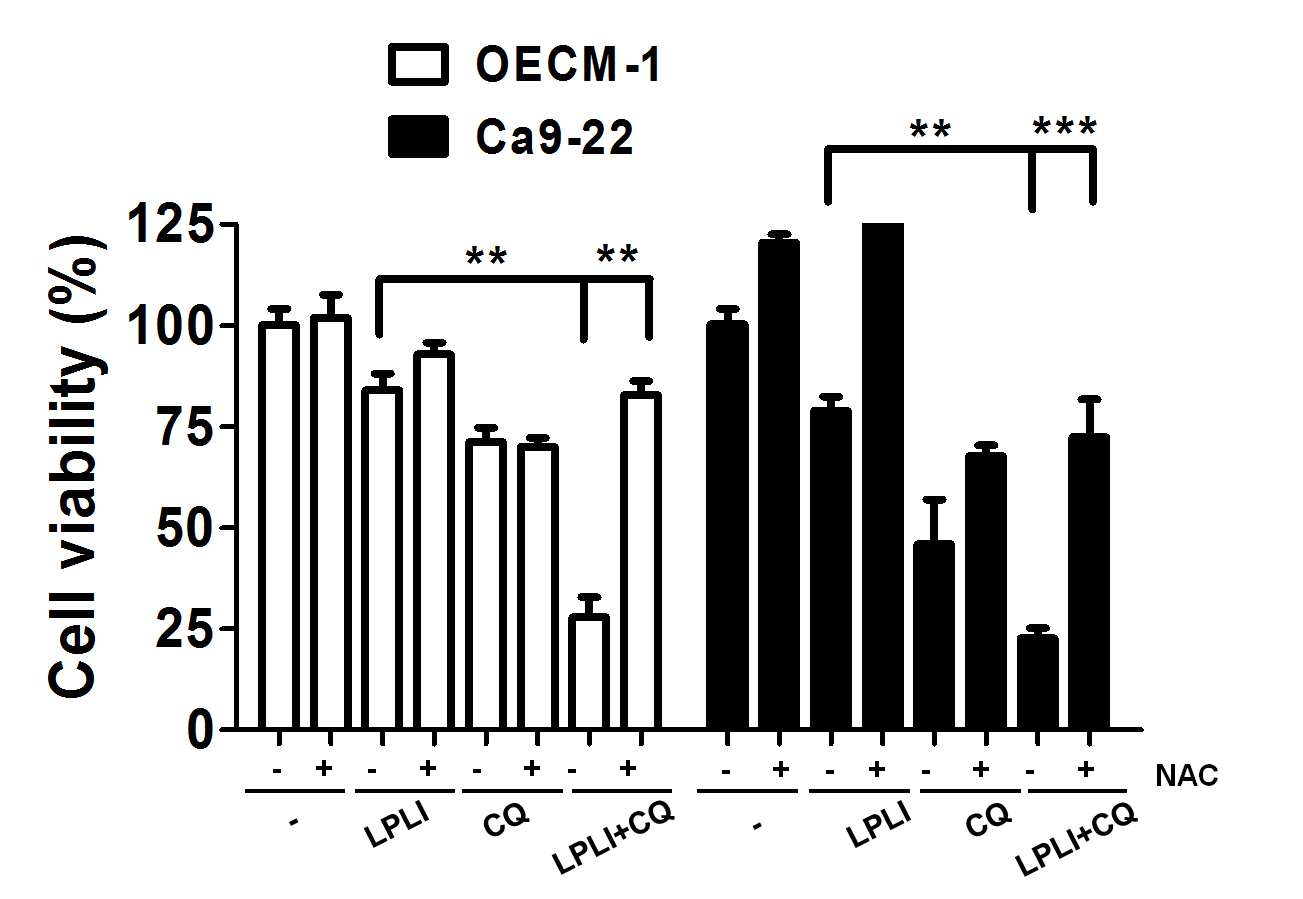

Supplement: S4 Fig — (TIF) [file pone.0160586.s004.tif]
